# Beyond Pain: The Effects of OnabotulinumtoxinA Therapy on Sensitization and Interictal Symptoms in Chronic Migraine

**DOI:** 10.3390/toxins16050203

**Published:** 2024-04-23

**Authors:** Paolo Alonge, Filippo Brighina, Simona Maccora, Laura Pilati, Salvatore Di Marco, Davide Ventimiglia, Bruna Maggio, Ivana Cutrò, Cecilia Camarda, Angelo Torrente

**Affiliations:** 1 Department of Biomedicine, Neuroscience and Advanced Diagnostics (Bi.N.D.), University of Palermo, 90127 Palermo, Italy; alongep95@gmail.com (P.A.); simonamaccora1@gmail.com (S.M.); laura.pilati.91@gmail.com (L.P.); dimarcosal@gmail.com (S.D.M.); davide.ventimiglia@unipa.it (D.V.); brnmaggio@gmail.com (B.M.); ivanacutro1993@gmail.com (I.C.); cecilia.camarda@unipa.it (C.C.); angelo.torrente@unipa.it (A.T.); 2 Neurology Unit, ARNAS Civico di Cristina and Benfratelli Hospitals, 90127 Palermo, Italy; 3 Neurology and Stroke Unit, P.O. “S. Antonio Abate”, 91016 Erice, Italy

**Keywords:** migraine, chronic migraine, onabotulinumtoxinA, central sensitization, interictal burden, interictal symptoms

## Abstract

Chronic migraine is a disease with a high burden on patients from both a working and quality of life point of view. The pathophysiology of this subtype of migraine is due to several factors, such as medication overuse. Nevertheless, the detrimental recurring of headache attacks with central and peripheral sensitization plays a central role and explains some additional symptoms complained about by these patients even in the interictal phase. OnabotulinumtoxinA is a therapy indicated for chronic migraine since it has proven to reduce peripheral sensitization, showing even efficacy on central symptoms. The aim of this narrative review is to present the current evidence regarding the effect of OnabotulinumtoxinA on sensitization and interictal symptoms.

## 1. Introduction

Chronic migraine (CM) represents a subclassification of migraine due to its deterioration and is defined as the presence of at least 15 headache days per month, of which at least 8 show migraine characteristics (or lead to anti-migraine acute drug intake) for at least 3 consecutive months [1]. The chronification (i.e., the increase in headache frequency leading to CM) of migraine, besides pain, leads to other consequences, such as an increased burden from associated symptoms, (e.g., nausea, photophobia, and phonophobia) and a greater incidence of comorbidities (e.g., cognitive symptoms, anxious and/or depressive symptoms, and medication overuse), which may cause disability even in migraine-free days (i.e., interictal phase) [2,3,4]. The increased incidence of such symptoms in CM compared to episodic migraine (EM) is reputed as an effect of central sensitization mechanisms [5]. Recently, the burden of migraine in interictal periods has been investigated more and more as it greatly affects the quality of life (QoL), especially in patients affected by CM, and it can even be modified by therapeutic interventions. OnabotulinumtoxinaA (BoNT-A) is a therapy specifically thought to counteract the peripheral sensitization in CM and may play a role even in the resolution of central sensitization symptoms. In the present work, we review the available evidence about the effect of BoNT-A on migraine beyond pain resolution.

## 2. Epidemiology and Burden of Chronic Migraine

### 2.1. Impact of Associated Symptoms

CM is a primary headache that affects approximately 2% of the population worldwide and shows significant associated morbidity. The disorder occurs more commonly in women than in men and presents the highest prevalence between 18 and 50 years of age. Worsening QoL, CM has a significant socioeconomic cost, with loss of working days and productivity both in paid and unpaid work (e.g., housekeeping activities) [6,7].

When considering the impact of chronic migraine, headache frequency and intensity are not the only relevant variables to take into account; associated symptoms, such as nausea, vomiting, photophobia, phonophobia, dizziness, vertigo, rhinorrhea, lacrimation, and osmophobia also contribute to the disabling nature of migraine. To value the severity of associated symptoms, gender must be taken into consideration since women reported greater severity of symptoms than men, namely pain intensity, nausea, photophobia, and phonophobia [8,9].

The impact of migraine characteristics on disability/QoL (Fn/QoL) often focuses on pain, while the impacts of associated symptoms, such as phonophobia and photophobia, have rarely been assessed thoroughly. Therefore, there is less information available about the impact of migraine-associated symptoms (nausea, vomiting, photophobia, and phonophobia) on Fn/QoL compared to the data on migraine frequency and severity of headache pain associated with impairments in Fn/QoL in several studies [8].

Furthermore, the relationship between pain and other associated symptoms has not been systematically evaluated in migraine attacks. However, in some studies, this relationship has been experimentally evaluated in migraine patients and controls. Drummond et al. in 2005 demonstrated that the painful stimulation of the temple induces nausea in migraineurs but not in healthy controls; moreover, after inducing nausea through optokinetic stimulation, pain perception increased. Given the strict relationship between nausea and pain intensity, it can be conjectured that migraine symptoms influence each other, producing a multiplier effect, resulting in a more intense and prolonged migraine attack [10].

### 2.2. Interictal Burden of Migraine

**Figure 1:**
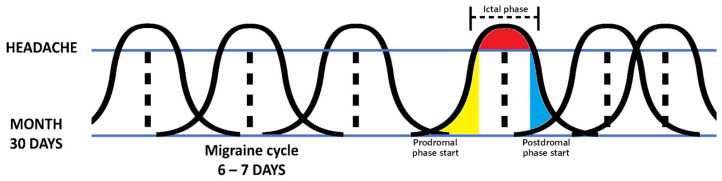
Simplification showing how, despite headaches not being present every day of the month in CM, the prodromal and postdromal phases often overlap and the interictal phase is hardly ever reached. Note that the bell curves represent just a simplification of a migraine with a 2 days prodromal phase, a 2–3 days headache phase, and 2 days postdromal phase. In the real world, every phase may show a different duration among the different cycles. Abbreviations: CM = chronic migraine.

Migraine shows a cyclical pattern of activity: the attack, or ictal phase, can last from 4 h to 3 consecutive days; moreover, many patients experience both premonitory symptoms before the acute attack (i.e., prodromal phase) and residual symptoms afterwards (i.e., postdromal phase). The period between two attacks (i.e., between a postdromal phase and the next prodromal one), constitutes the interictal phase [11]. Thus, migraineurs go through a premonitory phase, then they move on to the full-blown attack that includes a severe headache, which in turn is followed by a postdromal phase of symptoms decline and fatigue, and then back to the interictal phase [1,12]. However, studies have shown that the interictal phase should not be considered a period of complete remission and well-being, as both emotional and non-emotional (neurological) symptoms that characterize the acute phase of migraine can persist and impact QoL even between attacks. These include symptoms of hypersensitization, such as cutaneous allodynia, changes in taste and smell, photophobia, phonophobia, osmophobia, and changes in visual perception, as well as emotional symptoms such as depression, anxiety, fear, and worry about how a future attack may affect planned activities [2,13,14]. All these phenomena characterize the ‘interictal burden’. However, we must consider that the premonitory phase can occur up to 72 h before the ictal phase, the migraine attack can last up to 72 h and the prodromal phase can last up to about 24 h [15]. Therefore, in patients with CM, the prodromal and postdromal phases often overlap, and, for this reason, these patients may never reach the interictal phase (see Figure 1) [1,16].

Neurophysiological and imaging studies suggest that the brain of migraineurs shows both functional and structural peculiarities: migraine patients with interictal photosensitivity have thicker cortical regions in the right lingual region, cingulate isthmus, pericalcarine regions, left precentral, postcentral, and supramarginal regions. Changes in the activity of certain brain regions (i.e., increased activity in the inferior and superior occipital gyrus, pontine nuclei, and cerebellar lobules V and VI, and decreased activity in the middle frontal gyrus and cerebellar lobule VIIb) have been documented and correlated with motion sickness and disability scores, suggesting increased susceptibility to dizziness and motion sickness [16,17].

A recent pharmaceutical study reported that treatment with the anti-calcitonin gene-related peptide (anti-CGRP) monoclonal antibody (mAb) galcanezumab significantly reduced the migraine interictal burden, as measured by specific clinical scales [18,19]. Several studies have also shown that patients with EM or CM have elevated levels of CGRP in their blood and saliva in the interictal period. However, patients with CM who responded to BoNT-A treatment had reduced blood levels of interictal CGRP compared to those who did not respond to treatment [20]. Therefore, migraine burden should not be assessed only as a product of the severity and frequency of attacks. It is necessary to consider the impact of persistent symptoms that also affect the interictal phase. The Migraine Interictal Burden Scale (MIBS-4), was developed with the specific purpose of quantifying the interictal load of disability. The MIBS-4 is a self-administered questionnaire consisting of four items that allow for an assessment of the level of impairment in the family and social life of migraine patients at work or school, the presence of emotional/affective and cognitive distress, and the difficulty in being able to carry out plans or commitments in the last 4 weeks on days without headaches. When MIBS-4 was applied in a cross-sectional, observational, population-based (Overcome-Japan) web survey of Japanese with migraine, 41.5% of respondents experienced moderate to severe interictal burden that worsened with increasing frequency [11,21]. A recent pharmaceutical study reported that treatment with the anti-calcitonin gene-related peptide (anti-CGRP) monoclonal antibody (mAb) galcanezumab significantly reduced migraine interictal burden as measured by specific clinical scales [18,19]. Several studies have also shown that patients with EM or CM have elevated levels of CGRP in their blood and saliva in the interictal period. However, patients with CM who responded to BoNT-A treatment had reduced 147 blood levels of interictal CGRP compared to those who did not respond to treatment [21]. Therefore, assessment of interictal burden should be implemented not only in routine clinical practice but also in clinical trials as a measure to evaluate the efficacy of migraine medications. Therefore, the assessment of interictal burden should be implemented not only in routine clinical practice but also in clinical trials as a measure to evaluate the efficacy of migraine medications.

## 3. Pathogenesis of Chronic Migraine

### 3.1. Central and Peripheral Sensitization

A combination of peripheral and central sensitization appears to be implicated in the pathogenesis of migraine [22]. Central sensitization (CS) can be defined as an increased responsiveness of central neurons to afferent inputs with a normal or sub-threshold intensity. Peripheral sensitization (PS), on the other hand, regarding migraine, is due to an increased sensitivity of the trigeminovascular system sensory fibers involved in the cascade of events leading to pain [23,24]. Different diseases are related to CS: restless legs syndrome, chronic fatigue syndrome, fibromyalgia, temporomandibular joint disorder, migraine, tension-type headache, irritable bowel syndrome, and multiple chemical sensitivity [24]. Cutaneous allodynia (CA), the phenomenon when non-painful stimuli applied to normal skin evocate a pain perception, is present in 60% of patients with migraine and up to 90% with CM, both in the cephalic and extra-cephalic regions [25,26]. More studies relate the CA to a CS of trigeminovascular neurons, leading to a dysfunction of the trigemino–thalamo-cortical nociceptive pathway; those wider alterations of sensory stimuli processing may explain the phenomenon of extra-cephalic CA in CM [27]. An abnormal neuronal excitability in the TNC (trigeminal nucleus caudalis) was related to CS in CM in an animal model, and it is implicated in pain amplification [28,29]. On the basis of CS, there is an imbalance between the excitatory and inhibitory neurotransmitters, Glutamate/GABA, and the disequilibrium of monoamine neurotransmitters such as histamine and serotonin [30]. Plasma glutamate levels, a possible marker of CS, were higher in patients with CM and EM than those in controls [30,31]. Peripheral sensitization is also implicated in migraine chronification. In the arterial wall of patients affected by CM, Del Fiacco et al. found a significant increase in transient receptor potential vanilloid type-1 receptor (TRPV1), which evokes the release of CGRP and substance P, thus constituting the basis of a higher sensitivity to algogenic agents. BoNT-A can reduce TRPV1 in the rat trigeminal ganglion, suggesting a potential explanation for the high efficacy of BoNT-A in CM [32,33].

### 3.2. Medication Overuse Headache

Medication overuse headache (MOH) is a chronic headache disorder attributed to the frequent or regular use of acute medications in patients with a primary headache disorder. According to the current classification of the International Headache Society (ICHD-3), MOH is defined as a headache occurring for 15 or more days per month that develops because of regular overuse of medication for acute or symptomatic headache (use for >10 days per month or >15 days per month, depending on the medication) for more than 3 months, when the symptoms are not better described by another diagnosis [1].

The pathophysiology of MOH remains poorly understood but probably involves brain network alterations involved in chronification of pain, exposure to psychologic and socioeconomic factors, and genetic predisposition. The angiotensin converting enzyme (ACE) insertion or deletion polymorphism, the *BDNF* mutation Val66Met, or polymorphisms in catechol-O-methyltransferase (*COMT*) and serotonin transporter (*SLC6A4*) genes have been suggested as genetic risk factors in humans, but clear causal links are yet to be established [34,35,36]. These genes were associated with serotonergic and dopaminergic transmission, drug dependence, metabolic pathways, oxidative stress, and CGRP pathways.

Patients with migraine often show painful responses to normally innocuous sensory stimuli (e.g., touch, sound, and light), that elicit common symptoms during migraine attacks (i.e., allodynia, photodynia, and phonodynia). The allodynic phenomenon suggests the presence of neural amplification processes, which are referred to as sensitization. The overuse of acute pain-relieving medications (e.g., triptans, opioids) may induce sensitization due to increased evoked transmitter release, temporal summation, and expansion of receptive fields [37]. Conversely, the sudden interruption of acute medication use may lead to withdrawal-induced headaches. A preclinical study showed that after sustained exposure to morphine in rats, precipitation of withdrawal by microinjection of opioid antagonists within the rostral ventromedial medulla led to increased activity and expression of c-Fos in dura-sensitive spinal trigeminal nucleus caudalis neurons, as well as in the subnucleus reticularis dorsalis, an area linked to diffuse noxious inhibitory control [38]. States of sensitization probably promote vulnerability to the typical provocative stimuli associated with migraine attacks, such as stress and nitric oxide donors. Sumatriptan-sensitized rats also showed a lower threshold for cortical spreading depression [38,39,40]. Electrophysiological investigations have shown neuronal hyperexcitability with an increased stimulation response and a habituation deficit in patients with medication overuse headaches. These alterations have been observed with different stimulation techniques, such as somatosensory-evoked potentials [41,42], cold pressor tests [43], and laser CO_2_ evoked potentials [44], both in the cephalic and extracephalic regions [41]. Additionally, the hyperexcitability pattern in MOH seems to depend on the overused drug. Patients with this condition show an increased amplitude of somatosensory-evoked potentials when stimulating the median-nerve, but patients who overuse triptans show lower amplitudes than patients overusing NSAIDs [42]. The observed differences could reflect triptan-induced changes within the central serotonergic transmission. Patients overusing analgesics have lower 5-HT levels, higher 5-HT uptake, and higher 5-HT2A receptor density in blood platelets [45,46,47,48]. Imaging studies with different modalities have shown structural, functional, and metabolic changes of the brain in patients with MOH [49]. MOH patients have decreased grey matter volume in the orbitofrontal cortex compared with those without MOH; this finding is relevant because the orbitofrontal cortex is part of the mesocorticolimbic system implied in addictive behaviors [50]. Becerra et al., in a functional MRI study conducted on rats treated with sumatriptan, found differences in several resting state networks, including the default mode, autonomic, basal ganglia, salience, and sensorimotor networks; these differences were accompanied by cortical spreading depression-like phenomena [51]. CGRP may be involved in the sensitization process; asensitizedbb, an anti-CGRP antibody, blocks pain-like behaviors in rats sensitized with pain medications (sumatriptan or opioids) [51,52]. Even stress-elicited allodynia was prevented in sumatriptan-sensitized rats by blocking signaling at kappa opioid receptors (KOR) in the central nucleus of the amygdala [51,52,53]. Morphine-sensitized rats showed a loss of the diffuse noxious inhibitory controls with different stimulation modes, such as the sensory-evoked response that was restored by KOR antagonists in the right central nucleus of the amygdala [54]. These observations, both in humans and animal models, suggest that medications can promote sensitized states and alter the descending pain modulatory pathways, thus leading to pain facilitation and increased vulnerability to migraine triggers. InensitizeddIn sensitized states, enhanced dynorphin and KOR signaling in amygdala circuits might promote pain resulting from environmental stimuli; such evidence poses a biological basis for the mechanisms behind headache chronification induced by medication overuse. This might suggest a possible target for therapeutic interventions that aim to disrupt the vicious circle of medication overuse headaches.

## 4. Rationale for OnabotulinumtoxinA Preventive Therapy

CM is associated with disability and has a high impact on QoL, including health, social, and occupational functioning, so patients with CM require prophylactic treatment to prevent attacks and reduce headache frequency, severity, associated disability, and medication overuse [55]. First-line treatments include oral drugs such as beta-blockers, anticonvulsants, calcium-channel blockers, and tricyclic antidepressants. None of these drugs are primarily used for migraine treatment, and they all may provoke considerable side effects that can undermine compliance with treatment; moreover, there is no conclusive evidence that shows the superiority of one pharmacological class over the others [56]. Therefore, when choosing a preventing therapy, it is preferable to consider the patient’s comorbidities to reduce the risk of side effects and, when possible, to treat multiple conditions at once (e.g., choosing an anticonvulsant in patients affected both by epilepsy and migraine).

On the other hand, second-line treatments include medications with a more targeted effect on migraine mechanisms, such as anti-CGRP monoclonal antibodies and anti-CGRP small molecules, which have been specifically developed for migraine treatment, and BoNT-A injections [56].

OnabotulinumtoxinA contains 900-kDa of botulinum neurotoxin type A (BoNT-A), which is best known for its muscle paralysis effect, preventing the release of acetylcholine at the neuromuscular junction. So, it is usually used for blepharospasm, dystonia, and spasticity. In the management of migraines, onabotulinumtoxinA blocks the activation of nociceptive pathways [57]. It is used following the PREEMPT paradigm, which consists of 31–39 injections in some muscles of the head and neck [58], to be performed every 12 weeks. Usually at nerve terminals, small and big vesicles containing pro-inflammatory molecules, excitatory neurotransmitters, and neuropeptides are fused to the cell membrane through exocytosis and then recycling, or through a mechanism that includes the formation of a protein complex called the SNARE complex (soluble N-ethylmaleimide-sensitive fusion-attachment protein receptor), containing a protein called SNAP-25, the target of onabotulinumtoxinA. After the injection, the neurotoxin encounters the nerve terminals of C-fibers and thinly myelinated A-delta fibers of the trigeminal nerve and spinal cervical nerve, which are endocytosed by its heavy chain that binds a polysialoganglioside (PSG), so its light chain binds and cleaves SNAP-25 preventing the fusion of the big and small vesicles, containing glutamate, substance P, and CGRP that causes the pain and also decreases the CGRP high plasma level during the interictal phase of CM. This mechanism also decreases the insertion of transient receptor potential cation channel subfamily V member 1 (TRPV1) involved in propagation of the headache along the terminal nerves, confirming itself as an effective second-line prophylaxis therapy, reducing activation along peripheral and central pathways [57].

## 5. Effects on Central and Peripheral Sensitization Symptoms

**Figure 2:**
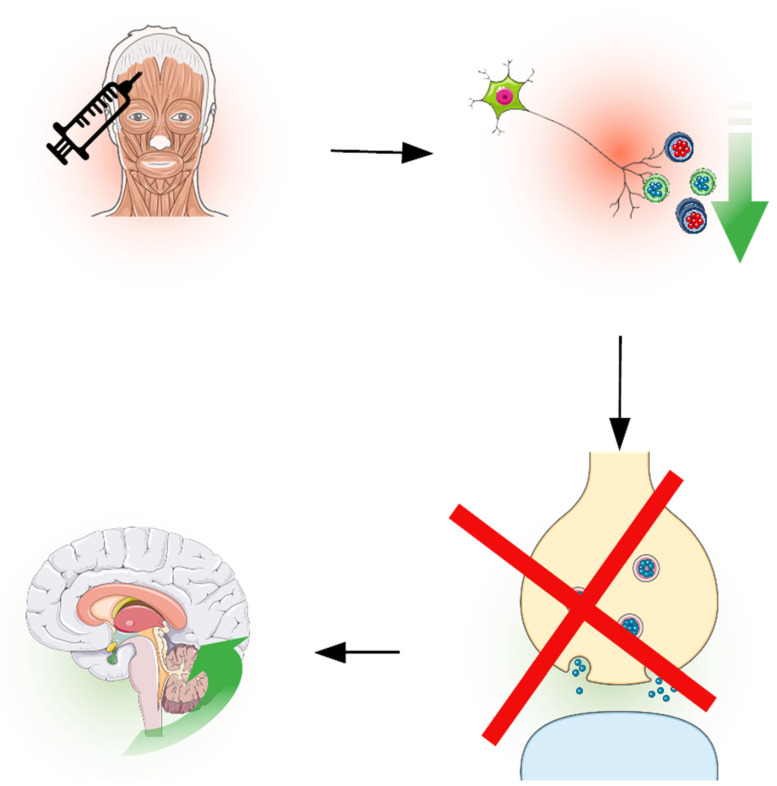
Mechanism of action of BoNT-A therapy on sensitization. The toxin, by blocking the SNARE complex protein SNAP25, reduces the release of pain mediators (e.g., CGRP, substance P). The reduced pain signaling from first order neurons produces an indirect effect on upper pain processing structures, which reduces central sensitization.

Several mechanisms have been postulated to elucidate the efficacy of onabotulinumtoxinA in CM. Pathogenetic studies have underlined the role of maladaptation of pain modulatory systems and subsequent sensitization of the trigeminal nociceptive pathway [5]. By interfering with SNARE-mediated trafficking and cleaving synaptosomal-associated protein-25 kDa (SNAP25), onabotulinumtoxinA may inhibit regulated exocytosis of vesicles containing neurotransmitters and proteins and block receptor insertion [59], resulting in a reduced liberation of pain mediators (e.g., substance P, CGRP, and glutamate) from primary nociceptive neurons and a decreased sensitization (see Figure 2). Moreover, onabotulinumtoxinA can reduce the membrane insertion of ion channels involved in nociception, such as transient receptor potential cation channel subfamily V member 1 (TRPV1), under sensitized conditions in meningeal C but not Aδ nociceptors [60,61]. All these mechanisms may be relevant to explain the indirect effects of BONT-A on central sensitization due to reduced pain signaling from sensory nerves (e.g., first-order sensory neurons located in cervical dorsal root ganglia 2 and 3 and trigeminal ganglia) to the spinal trigeminal nucleus [57]. Experimental studies have pointed out the possibility that BoNT-A can also have direct central effects on pain nociception. In the formalin-induced pain model, cleaved SNAP-25 was found in the trigeminal nucleus caudalis; moreover, the effects of onabotulinumtoxinA were completely abolished by colchicine, supporting the hypothesis that BoNT-A follows retrograde axonal transport and cell-to-cell transfer, or transcytosis [62]. The central antinociceptive effects of onabotulinumtoxinA can also rely on the enhancement of the endogenous opioid system, though the exact mechanisms of its action have not been cleared out yet [63]. The clinical counterpart of central sensitization is represented by allodynia, an abnormal sensory state in which an innocuous sensory stimulus is felt as painful and involves second-order neurons located in the caudal trigeminal nucleus. de Tommaso et al. have already observed that BoNT-A can reduce allodynia in chronic migraine patients after up to 2 years of treatment [64]. A more recent study by Ozarslan et al. confirmed that onabotulinumtoxinA can decrease allodynia as assessed by the average allodynia symptom checklist (ASC-12) but does not modify thermal thresholds evaluated by Quantitative Sensory Testing (QST) [65]. Psychiatric comorbidities associated with CM can also be modulated by BoNT-A, as demonstrated by several studies observing an improvement of anxiety and depressive symptoms after treatment. Such effects, which appear to be independent from migraine improvement, may be due to an indirect modulation of the limbic system [66]. Effects on sleep quality are instead controversial [67]. The clinical effects of BoNT-A on central sensitization syndromes are also experienced in other clinical conditions characterized by chronic pain. For instance, in complex regional pain syndrome (CRPS), a reduction of myofascial pain and allodynia was achieved after BoNT-A injections [68]; similar results have already been observed in low back pain [69] and overactive bladder [70].

## 6. Effects on Interictal Symptoms and Comorbidity

The impact of BoNT-A treatment on interictal symptoms and comorbidities in migraineurs was extensively examined and the major studies are summarized in Table 1. The COMPEL study, conducted on 715 patients over 108 weeks to specifically assess the effects of BoNT-A therapy on comorbid symptoms, found a clinically significant improvement in anxiety and depression symptoms, sleep quality, and fatigue; moreover, the improvement increased gradually over the observation period [66]. Aydinlar et al., in a single-center prospective study that evaluated 190 patients treated with BoNT-A over 48 weeks, found that sleep quality improved only in patients without negative emotional states at baseline, while patients with relevant depression and/or anxiety symptoms did not improve either in sleep quality or negative emotional states, despite significantly decreased migraine frequency [71]. However, a recent meta-analysis conducted on 259 studies concluded that BoNT-A treatment improves the disease severity of both CM and major depressive disorder in patients with both diseases [4]. The gathered evidence suggests that BoNT-A treatment improves mood and sleep indirectly by reducing headache days; moreover, comorbid depression and anxiety do not influence the efficacy of the therapy. Therefore, in patients with CM and mood disorders, BoNT-A should be preferred to other medications that can negatively affect mood (e.g., propranolol, anticonvulsants) or that perform poorly in this specific population (e.g., anti-CGRP monoclonal antibodies) [72]. Temporomandibular disorder (TMD) symptoms also show a higher prevalence among migraineurs compared to the general population; BoNT-A injections have been used as a treatment for TMDs targeting the masseter muscle and the anterior temporalis muscle, which is also treated in the PREEMPT protocol [58,73]. To date, only one trial has investigated the effectiveness of single and concomitant treatment of migraine and TMD in women with the comorbidity; the investigators found a significant improvement in both conditions only in the group that received both the migraine prophylaxis (propranolol 90 mg) and the TMD treatment (stabilization splint) compared to the other three groups (propranolol placebo, propranolol and non-occlusal splint, propranolol placebo and non-occlusal splint), and they concluded that in patients with the comorbidity, only the treatment of both conditions is effective [74]. However, the possible effectiveness of the PREEMPT protocol on TMD comorbid symptoms has not been investigated properly. In 2018, Kocaman et al. found that the presence of TMD symptoms did not constitute a predictive factor for response to BoNT-A treatment in a population of 30 patients with CM [74,75]. The possible mechanism of action of BoNT-A on comorbid TMD and migraine has been investigated by Lacković et al. on an animal model. They theorized that inflammation of the temporomandibular joint (TMJ) may cause a trigeminal sensitization that favors the chronicization of migraine. The study showed that CFA (complete Freund’s adjuvant)-evoked TMJ inflammation was accompanied by inflammatory changes in the cranial dura (plasma protein extravasation and inflammatory cell infiltration) and increased levels of CGRP; moreover, following peripheral toxin injection, cleaved SNAP-25 was colocalized with CGRP-expressing dural afferents, a sign of BoNT-A activity at this level. BoNT-A prevented the CFA-evoked dural inflammation and CGRP peptide increase in the cranial dura [76]. While far from conclusive, these studies provide interesting clues for further investigations of BoNT-A effects on comorbid migraine and TMD. Regarding interictal burden, a recent study conducted on 70 patients treated for three consecutive cycles (nine months) showed a significant reduction of interictal symptoms and cutaneous allodynia, assessed through MIBS-4 and ASC-12. Of interest, interictal burden improvement showed no relations to the persistence of allodynia; the authors concluded that this finding suggests a complex nature of interictal burden, which is not a simple sum of bothersome symptoms and that requires specific instruments to be assessed [77].

## 7. Conclusions

In the era of tailored therapies, the impact on interictal burden cannot be ignored in the choice of a preventive migraine medication; the classical drug choice method based on patients’ comorbidities and contraindications to pharmacological classes cannot be further considered satisfying. OnabotulinumtoxinA therapy is highly effective on pain intensity and frequency. Moreover, response to treatment is not influenced by other comorbidities (e.g., psychiatric disorders, fibromyalgia) or interactions with other drugs, which constitutes a remarkable strength in comparison to other migraine prophylaxes. Moreover, BoNT-A therapy shows promising effects on associated symptoms, typical migraine comorbidities, and interictal symptoms. Namely, the treatment improves mood and sleep, alleviates cutaneous allodynia, and may also influence dural inflammation in TMDs. However, further studies that directly assess such effects are needed to better define the role of BoNT-A treatment beyond pain resolution.

## Figures and Tables

**Table 1:** Summary of studies that investigated the effect of BoNT-A therapy on interictal symptoms and comorbidities.

Study	Number of Patients	Investigated Symptoms	Main Findings
Blumenfeld et al. [66]	716	Anxiety and depression, sleep quality, and fatigue	Significant improvement in all the investigated symptoms
Aydinlar et al. [71]	190	Sleep quality	Improvement only in patients without comorbid psychiatric disorders
Kocaman et al. [75]	30	TMDs influence on efficacy of BoNT-A migraine prophylaxis	No influence on efficacy
Argyriou et al. [77]	70	Interictal symptoms and cutaneous allodynia	Both symptoms improved

TMDs: temporo-mandibular disorders.

## Data Availability

No new data were created or analysed in this study. Data sharing is not applicable to this article.
